# Mesenchymal stem cells differentially affect the invasion of distinct glioblastoma cell lines

**DOI:** 10.18632/oncotarget.16041

**Published:** 2017-03-09

**Authors:** Barbara Breznik, Helena Motaln, Miloš Vittori, Ana Rotter, Tamara Lah Turnšek

**Affiliations:** ^1^ Department of Genetic Toxicology and Cancer Biology, National Institute of Biology, 1000 Ljubljana, Slovenia; ^2^ International Postgraduate School Jozef Stefan, 1000 Ljubljana, Slovenia; ^3^ Department of Biochemistry, Faculty of Chemistry and Chemical Engineering, University of Ljubljana, 1000 Ljubljana, Slovenia

**Keywords:** glioblastoma multiforme, proteases, mesenchymal stem cells, tumor heterogeneity, zebrafish model

## Abstract

Glioblastoma multiforme are an aggressive form of brain tumors that are characterized by distinct invasion of single glioblastoma cells, which infiltrate the brain parenchyma. This appears to be stimulated by the communication between cancer and stromal cells. Mesenchymal stem cells (MSCs) are part of the glioblastoma microenvironment, and their ‘cross-talk’ with glioblastoma cells is still poorly understood. Here, we examined the effects of bone marrow-derived MSCs on two different established glioblastoma cell lines U87 and U373. We focused on mutual effects of direct MSC/glioblastoma contact on cellular invasion in three-dimensional invasion assays *in vitro* and in a zebrafish embryo model *in vivo*. This is the first demonstration of glioblastoma cell-type-specific responses to MSCs in direct glioblastoma co-cultures, where MSCs inhibited the invasion of U87 cells and enhanced the invasion of U373. Inversely, direct cross-talk between MSCs and both of glioblastoma cell lines enhanced MSC motility. MSC-enhanced invasion of U373 cells was assisted by overexpression of proteases cathepsin B, calpain1, uPA/uPAR, MMP-2, -9 and -14, and increased activities of some of these proteases, as determined by the effects of their selective inhibitors on invasion. In contrast, these proteases had no effect on U87 cell invasion under MSC co-culturing. Finally, we identified differentially expressed genes, in U87 and U373 cells that could explain different response of these cell lines to MSCs. In conclusion, we demonstrated that MSC/glioblastoma cross-talk is different in the two glioblastoma cell phenotypes, which contributes to tumor heterogeneity.

## INTRODUCTION

Tumor heterogeneity is recognized as one of the key reasons for ineffective radiotherapy, chemotherapy and recurrence in many cancers, as occurs in the most aggressive glioma stage WHO IV, glioblastoma multiforme (GBM). GBMs are classified according to their gene-expression patterns, as the proneural, neural, classical and mesenchymal subtypes [1, 2]. Clonal evolution *via* GBM stem-like cells gives rise to heterogeneous populations of differentiated, invasive GBM cells [3]. We demonstrated that the generally used GBM cell lines, U87 and U373, show distinct phenotypes and they differ in expression of genes associated with extracellular matrix (ECM) organization, developmental processes and cell differentiation [4]. Upon the *in vitro* co-culturing of U87 and U373 cells, we identified different clusters of de-regulated genes in these two GBM cell lines. The molecular ‘cross-talk’ between U87 and U373 cells strikingly increased the invasiveness of both cells types [4], reflecting the mutually induced phenotypic changes, as may occur in tumors *in vivo*, and as has been suggested recently by *Ricklefs et al*. [5].

Cell invasion is a crucial obstacle to overcome in glioma treatment, as GBM cells represent distinct moving targets, spreading into various sites in the brain. Many studies have been focused on therapeutic targeting of various types of proteases involved in GBM cell invasion [6]. According to the MEROPS database (http://www.merops.ac.uk), the protease superfamily consists of 990 known and putative genes. Verbovšek *et al*. [7] used a transcriptomics approach that revealed 311 protease genes that were differentially expressed in both GBM tissues and in GBM U87 and U373 cell lines, when compared to normal brain and normal astrocytes, respectively. These proteases induce protease signaling [8] on their own, or in cascade-like reactions [9], which play important roles in GBM progression [6, 10, 11]. Most relevant players in pericellular invasion of GBM cells are distinct lysosomal cathepsins, such as cathepsins B and S [12–15], calpains [16], urokinase-type plasminogen activator and selected matrix metalloproteases [9–11], that enhance cell invasion [6, 9]. These proteases are considered as potential targets for anti-invasive therapy [10] and were selected in this GBM heterogeneity study.

Recent findings have suggested that cancer–stromal interactions contribute to a complex proteolytic network, that is regulated also *via* pro-inflammatory cytokines and a selection of growth factors [10, 17, 18]. Communication between stromal cells and GBM cells creates a tumor-promoting environment [19]. Stromal mesenchymal stem cells (MSCs) can induce the transition to a more invasive GBM cell phenotype [20] that shows similarities with epithelial to mesenchymal transition (EMT) or with the mesenchymal to amoeboid transition [21]. The key difference between these two cell migration modalities are proteases that are involved in cell invasion in co-culture models, as we showed in the present study.

MSCs are known as adult stem cells, and reside in many organs for the regeneration of damaged tissue. MSCs are increasingly used in cell therapies and tissue engineering because of their availability, multi-potency, and immunomodulatory activity [22]. Recruited bone marrow-derived MSCs can home neoplasia and become part of the tumor microenvironment [23–26], including GBM [27], but seem to have dual roles in tumors, which mainly depend on their immuno-activation status [28]. In glioma, both roles of MSCs in promotion [24, 26] and inhibition of tumor growth have been reported [26–30]. However, the molecular mechanisms of their interactions with GBM cells are not yet well defined.

To study tumor heterogeneity, we used a three-dimensional (3D) spheroid model, which included direct MSC/GBM cell contact, as well as paracrine signaling. As the critical step in translational oncology remains the bridge between *in vitro* cell cultures to *in vivo* animal models, as a preclinical phase I step, in this study we used a zebrafish model for that respect. The strongest benefit for their use as a tumor model is the transparency of the embryos that allows imaging of tumor progression at single-cell resolution in a real time [31–34].

The major aim of the present study was to determine how cross-talk between the phenotypically distinct GBM cell lines U87 and U373 and the bone marrow-derived MSCs mutually affects cell invasion. Using transcriptome analyses, we have identified the key upregulated proteases in GBM, and have revealed their differential expression in the two distinct GBM phenotypes, when in direct co-cultures with MSCs, which the protease expression was also altered. We have been also able to translate this *in-vitro* 3D model into the experimental *in vivo* zebrafish embryo model.

## RESULTS

### MSCs reduce invasion of U87 cells and enhance invasion of U373 cells *in vitro*

**Figure 1:**
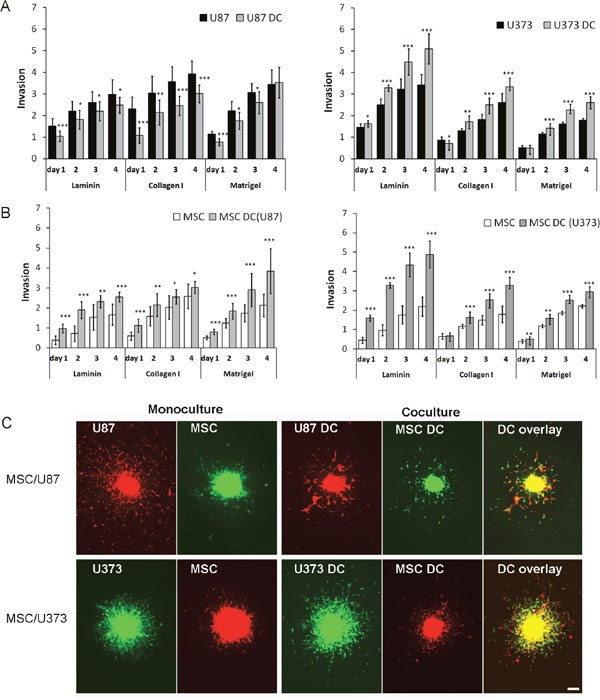
Invasion of DiO/DiI-labelled MSCs, U87dsRed and U373eGFP cells from spheroids Spheroids of MSCs, GBM cells (monocultured) and MSC/GBM cells as direct co-cultures (DC) were prepared and embedded in laminin, collagen I and Matrigel. Invasion distance *versus* spheroid diameter (Invasion) was measured over a period of 4 days using a fluorescent inverted microscope. **(A)** Invasion of U87 cells (left) and U373 cells (right) from spheroids. **(B)** Invasion of MSCs from spheroids, as MSCs co-cultured with U87 cells (left) and U373 cells (right). **(C)** Representative images of MSCs and U87 and U373 cells invading from monocultures and MSC/GBM direct co-cultures (DC) after 2 days in collagen I (magnification, 40×). Scale bar = 200 μm. Data are means ± SD. * P <0.05, ** P <0.01, *** P <0.001.

To determine the effects of MSCs and GBM cells on cellular invasion *in vitro*, we performed a 3D spheroid invasion assays, using various ECM components, such as laminin, collagen type I, and Matrigel. In monocultured spheroids, U87 cells were more invasive than U373 cells when embedded in collagen type I or Matrigel. In contrast, U373 cells were more invasive on laminin (Figure 1A). In the MSC/U87 spheroid co-cultures, U87 cells were significantly less invasive compared to U87 monospheroids, irrespective of the ECM component used (Figure 1A, left panel; Figure 1C). The strongest inhibition of U87 cellular invasion by MSCs was observed in collagen type I (up to 50% inhibition on day 1). In contrast, MSCs enhanced invasion of U373 cells compared to U373 monocultured spheroids by nearly 30% in collagen type I and by 45% in Matrigel (on day 4), whereas on laminin the highest increase in U373 cell invasion (50% on day 4) was observed (Figure 1A, right panel; Figure 1C). Both GBM cell lines enhanced the invasiveness of MSCs. This effects were dependent on the ECM component present, as U87 cells enhanced MSC invasion by 80% in Matrigel on day 4 (Figure 1B, left panel), whereas U373 enhanced invasion of MSCs by as much as 200% on laminin on day 3 and 4 (Figure 1B. right panel).

### Transcriptome analyses of GBM reveals upregulated protease genes

As cross-talk between MSCs and GBM cells altered invasive behavior of the GBM cells, we searched for proteases involved in the interplay between these 2 cell types. In a related study [7], we have investigated the deregulated GBM transcriptome of protease genes, as compared to that of normal brain tissue. Among the upregulated genes, we searched for intersections with a list of human proteases obtained from the MEROPS database, which resulted in 78 upregulated proteases in GBM *versus* normal brain, including 25 serine, 23 cysteine, 20 metallo-, 1 aspartic, and 3 mixed protease family genes (Supplementary Table 1). We selected seven proteases and one protease receptor for validation in the co-culture model, as this panel of proteases has been reported to be functionally associated with cellular invasion [6, 9].

### Validation of selected cysteine, serine and metallo-proteases in MSC/GBM co-cultures

**Figure 2:**
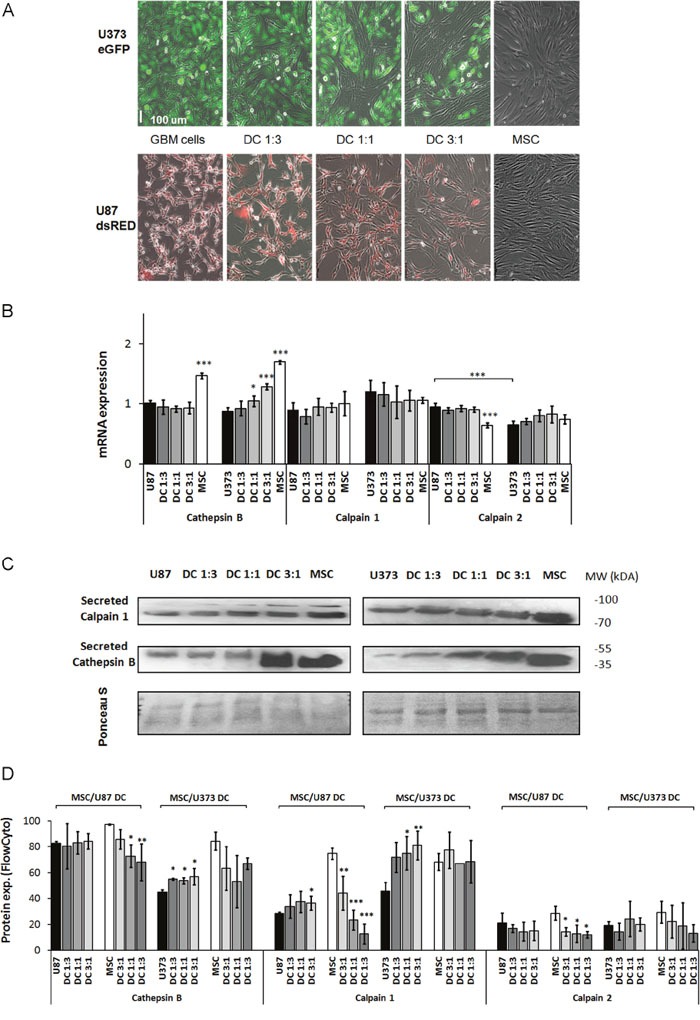
Expression of the cysteine proteases cathepsin B, calpain1 and calpain2 in GBM cells, MSCs and their direct co-cultures **(A)** MSCs and GBM cells (U87, U373) monocultured or in direct co-cultures (DC) at 3 different ratios, were grown for 72 h as monolayers and analyzed. **(B)** Cathepsin B, calpain1 and calpain 2 mRNA levels in U87 cells, U373 cells, MSCs, and in their direct co-cultures (DC) as determined by qPCR. **(C)** Detection of secreted cathepsin B and calpain1 in culture medium of MSCs, U87 cells (left), U373 cells (right) and their co-cultures (DC) at different ratios, using Western blotting. Western blots were stained with Ponceau S to confirm equal protein loading. **(D)** Cathepsin B, calpain1 and calpain2 protein expression in MSCs and GBM cells grown as monocultures and direct co-cultures at different ratios, analyzed by flow cytometry. Data are means ± SD. * P <0.05, ** P <0.01, *** P <0.001.

The canonical proteolytic cascade that leads to enhanced cancer cell invasion is considered to be initiated by (pro)cathepsin B, which translocates from lysosomes to plasma membranes, where it may activate uPA, which in turn activates MMP-14, followed by activation of gelatinases MMP-2 and MMP-9 [14]. We analyzed gene expression of these proteases using RT-PCR and protein levels by Western blotting and flow cytometry, in media and cells of monocultures and MSC/GBM co-cultures. To demonstrate the impact of MSC:GBM cell ratios on protease expression in these cells, we used 3 ratios of MSCs to GBM cells, i.e., 1:3, 1:1 and 3:1 (Figure 2A).

Changes in cathepsin B mRNA content was not observed in the MSC/U87 co-cultures, whereas cathepsin B mRNA was upregulated by 20-45% in MSC/U373 direct co-cultures in a cell-ratio-dependent manner when compared to the respective monocultures (Figure 2B). Increased cathepsin B protein secretion was observed only in the 3:1 ratio of MSC/U87 co-cultures (Figure 2C, left panel) and in all MSC:GBM ratios of MSC/U373 co-cultures (Figure 2C, right panel). Flow cytometry confirmed no alteration of cellular cathepsin B protein in co-cultured U87 cells, but upregulation of cathepsin B protein expression in co-cultured U373 cells at all MSC:GBM ratios by 20%. In contrast, both GBM cell types downregulated cathepsin B expression in MSCs by 15-30% (Figure 2D).

mRNA levels of calpain1 and calpain2 did not change in either of the MSC/GBM co-cultures (Figure 2B). Secreted calpain1 increased only slightly in MSC/U87 co-cultures with 3:1 ratio, and in all MSC/U373 co-cultures. Increased calpain1 in co-cultures was most likely secreted from GBM cells (Figure 2C), as MSCs upregulated protein expression of calpain1 only slightly in the co-cultured U87 cells with 3:1 ratio and by up to 80% in the co-cultured U373. In contrast, calpain1 was significantly downregulated in the co-cultured MSCs with U87 cells and not altered in co-cultured MSCs with U373 cells. Calpain2 protein levels were lower in both GBM cell, lines and not detected in the media, whereas in MSCs calapin2 was downregulated in MSCs in co-cultures with U87 cells, but not with U373 cells (Figure 2D).

**Figure 3:**
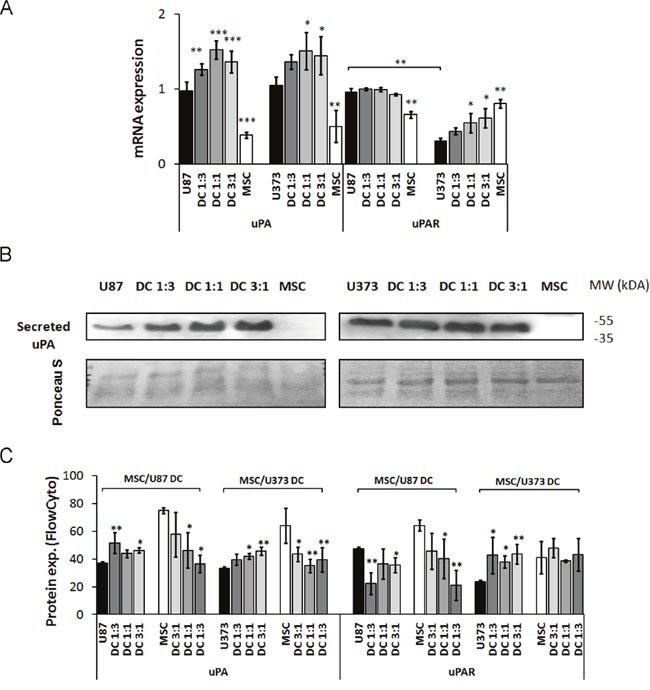
Expression of serine protease urokinase-type plasminogen activator (uPA) and its receptor (uPAR) in GBM cells, MSCs and their co-cultures (DC) MSCs and GBM cells were mixed in different ratios, grown for 72 h as monolayers and analyzed. **(A)** uPA and uPAR mRNA levels in U87 cells, U373 cells, MSCs and their direct co-cultures at different ratios, as determined by qPCR. **(B)** Detection of uPA secreted into the cell medium of MSCs, U87 cells (left), U373 cells (right) and their co-cultures (DC) at different ratios, as analyzed by Western blotting. The Western blots were stained with Ponceau S to confirm equal protein loading. **(C)** uPA and uPAR expression analyzed in GBM cells and MSCs grown as monocultures and co-cultures at different ratios, as analyzed by flow cytometry. Data are means ± SD. * P <0.05, ** P <0.01, *** P <0.001.

mRNA levels of the serine protease uPA increased by 30-50% in both MSC/U87 and MSC/U373 co-cultures, as compared to monocultures (Figure 3A). The increased secretion of uPA from both of these co-cultures was confirmed by Western blotting (Figure 3B), and increased protein expression in the co-cultured GBM cells was confirmed by flow cytometry (Figure 3C). In at contrast, uPA protein expression decreased in MSCs co-cultured with either of these GBM cell lines subtype by more than 30% at 1:3 ratio (Figure 3C). mRNA levels of the urokinase receptor uPAR remained unchanged in MSC/U87 co-cultures, when compared to monocultures, but increased by more than 40% in MSC/U373 co-cultures (in DC 3:1), when compared with monocultures (Figure 3A). uPAR protein levels decreased in co-cultured U87 cells by 20%, but increased by 60-80% in co-cultured U373 cells. In contrast, MSCs when co-cultured with U87 cells, showed decrease in uPAR expression in a cell-ratio-dependent manner (30-70%), whereas when co-cultured with U373 cells uPAR expression remained unchanged (Figure 3C).

**Figure 4:**
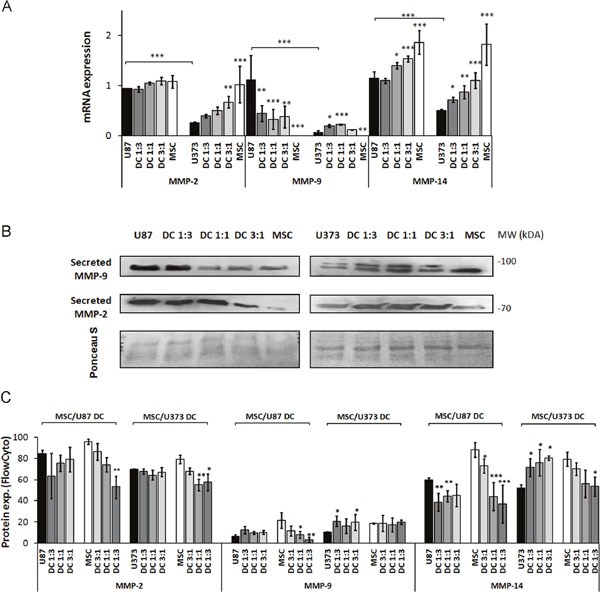
MMP-2, MMP-9 and MMP-14 expression in GBM cells, MSCs and their co-cultures MSCs and GBM cells were monocultured or direct co-cultured (DC) at different ratios, grown for 72 h as monolayers and analyzed. **(A)** MMP-2, -9 and -14 mRNA level, as determined by qPCR. **(B)** Secreted MMP-2 and MMP-9 in culture medium of MSCs, U87 cells (left), U373 cells (right) and their co-cultures, by Western blotting. The western blots were stained with Ponceau S to confirm equal protein loading. **(C)** MMP-2, -9 and -14 expression, analyzed by flow cytometry. Data are means ± SD. * P <0.05, ** P <0.01, *** P <0.001.

mRNA levels of metalloprotease MMP-2 in MSC/U87 co-cultures did not change (Figure 4A), and alteration in MMP-2 secretion was not detected in MSC/U87 co-cultures, as compared to U87 monocultures, except for decrease in the 3:1 co-cultures (Figure 4B, left panel). mRNA levels of MMP-2 increased in MSC/U373 co-cultures up to 50% in 3:1 co-cultures, as compared to U373 monocultures (Figure 4A). This was in line with increased secretion of MMP-2 protein from MSC/U373 co-cultured cells (Figure 4B, right panel). Flow cytometry revealed no changes in MMP-2 protein expression in either of the GBM cell types co-cultured with MSCs. In contrast, MSCs showed a cell-ratio-dependent decrease in MMP-2 expression in both types of co-cultures (Figure 4C).

MMP-9 mRNA levels were decreased in MSC/U87 co-cultures, but increased 2.5-fold in MSC/U373 co-cultures in 1:1 and 1:3 ratios, as compared to their respective monocultures (Figure 4A). This coincided with secreted MMP-9 protein, which was decreased in MSC/U87, but increased in MSC/U373 co-cultures (Figure 4B). This was in line with the unchanged protein expression of MMP-9 in co-cultured U87 cells, and increased MMP-9 expression up to 100% in co-cultured U373 cells in 1:3 and 3:1 ratio (Figure 4C). Although MMP-9 protein expression did not change in the co-cultured U87 cells, the decreased MMP-9 secretion by MSC/U87 co-cultures may have been the result of the downregulation of MMP-9 protein expression in the co-cultured MSCs by up to 85% in 1:3 co-cultures (Figure 4C).

Interestingly, MMP-14 mRNA increased by up to 30% in MSC/U87 co-cultures, and also by up to 110% in 3:1 MSC/U373 co-cultures (Figure 4A). However, MMP-14 protein expression was downregulated by up to 30% in co-cultured U87 cells, whereas it was upregulated in co-cultured U373 cells, by 40-55% in 1:3, 1:1 and 3:1 ratios, respectively (Figure 4C). In contrast, MMP-14 protein expression in MSCs was decreased by 25-60% in U87 co-cultures, and by 30-35% in U373 cell co-cultures.

**Figure 5:**
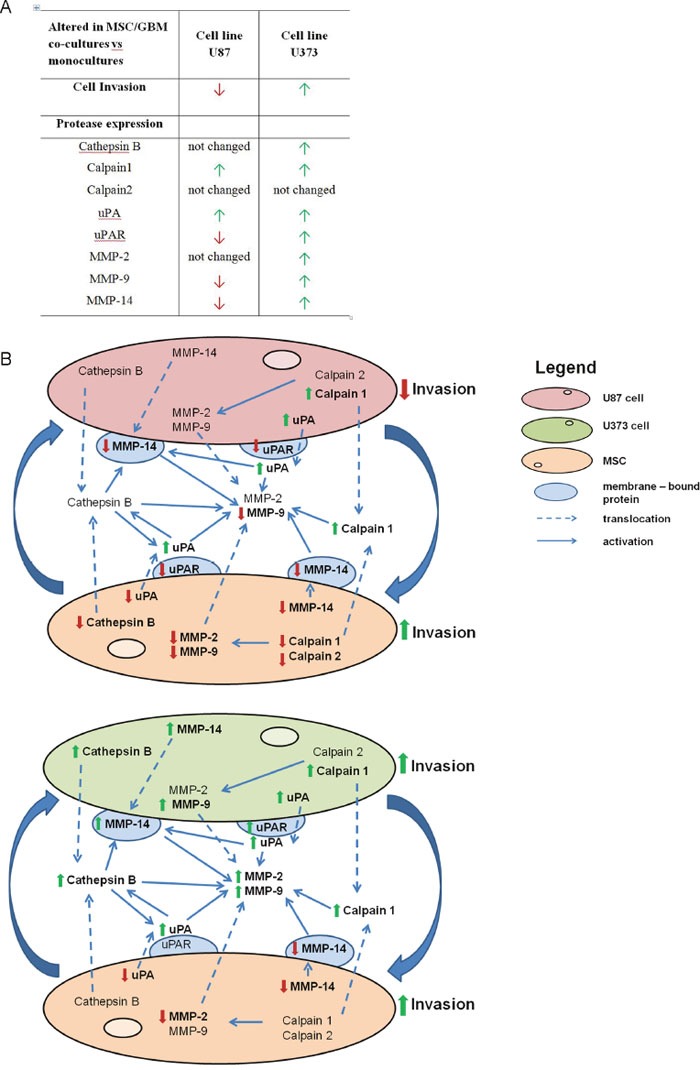
Alterations in invasion and the protease expression in GBM cells and MSCs upon co-culturing **(A)** Changes in invasiveness and the candidate protease expression in GBM cells after co-culturing with MSCs are presented in the table. Red and green arrows indicate lower and higher invasion of GBM cells from MSC/GBM co-cultures, respectively, compared with monocultures. Similarly, red and green arrows indicate decreased and increased expression, respectively, at any of the levels of the candidate proteases, compared with monocultures. **(B)** The scheme shows the presentation of the effects of MSC/GBM co-culturing on the protease expression, involved in invasion-associated proteolytic cascade, in the respective cells and their co-culture media. The upper part of the scheme represents direct cross-talk of U87 cells with MSCs, resulting in decreased invasion of U87 cells, correlating with decreased expression of uPAR and MMP-14 and secretion of MMP-9. Simultaneously, MSC invasion was increased in spite of decreased expression of all the proteases. The lower part of the scheme shows that enhanced U373 invasion was associated with increased levels of all proteases, except calpain2. This shows that MSCs trigger opposite response of two types of GBM cells with respect to invasion, which is associated with different pattern of proteases expression. The lowest part of the scheme shows that MSC/U373 cell cross-talk also results in increased MSC invasion, downregulation of uPA, MMP-9 and -14 and not altered expression of other tested proteases. Alterations of invasion and proteases are shown as bold red arrows (down-regulation) and green arrows (up-regulated).

Taken together, cathepsin B, calpain1, and MMP-2, -9, and -14 were upregulated at the mRNA and/or protein levels in U373 cells, co-cultured with MSCs that showed enhanced invasion. However, among tested proteases only the protein expression of MMP-14, which was downregulated in co-cultured U87 cells, correlated with decreased invasion of U87 cells upon co-culturing with MSCs. uPA was upregulated in both of these cell lines, although its receptor was upregulated in U373 and downregulated in U87 cells in co-cultures. In MSCs, both types of GBM cells downregulated most of the above described proteases (Figure 5).

### Invasion of GBM cells from MSC/GBM co-cultures is differentially affected by protease inhibitors

**Figure 6:**
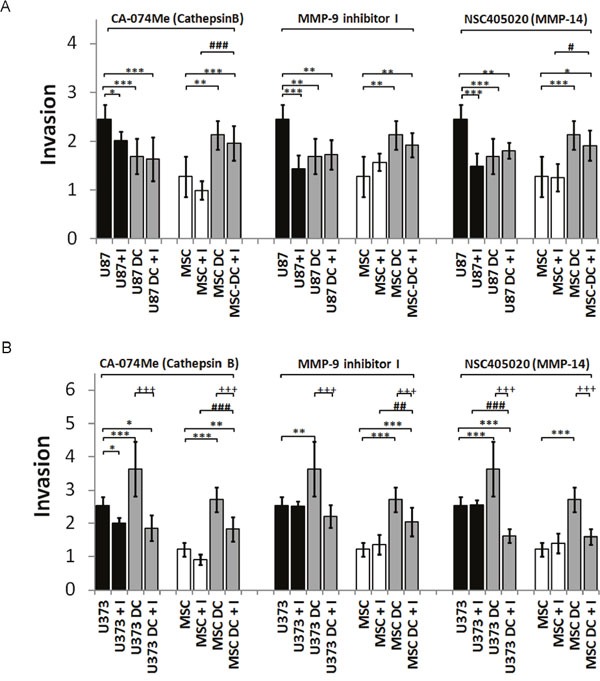
Invasion of DiO/DiI-labelled MSCs and GBM (U87 dsRED, U373 eGFP) cells from spheroids upon treatment with selective inhibitors of cathepsin B (2 μM), MMP-9 (100 nM) and MMP-14 (10 μM) Generated monospheroids (MSCs, U87, U373) and mixed spheroids were incubated in laminin-coated wells and treated with protease inhibitors or control (0.1% DMSO). **(A)** Invasion (invasion distance/ spheroid diameter) of MSCs and U87 cells from spheroids in the presence of protease inhibitors or control medium, measured after 72 h. **(B)** Invasion of MSCs and U373 cells from spheroids in the presence of protease inhibitors or control medium after 72 h. Data are means ± SD. * P <0.05, ** (++, ##) P <0.01, *** (+++, ###) P <0.001.

We performed spheroid invasion assays in the presence and absence of selective synthetic inhibitors of cathepsin B, MMP-9 and MMP-14: CA-074Me, MMP-9 inhibitor I, and NSC405020, respectively, at non-toxic concentrations (Supplementary Figure 1). In U87 monocultured spheroids, cell invasion was inhibited by 20% by the cathepsin B inhibitor, by 40% by the MMP-9 inhibitor, and by 42% by the MMP-14 inhibitor (Figure 6A). Invasion of U87 cells from the MSC/U87 co-cultured spheroids was already inhibited, and no further changes were observed upon addition of any of the inhibitors. The protease inhibitors had no effects on invasion of MSCs from MSC/U87 spheroids (Figure 6A). In contrast, only CA-074Me reduced invasion of U373 cells from monospheroids by 20%, whereas both the cathepsin B and MMP-9 inhibitors reduced U373 cell invasion from spheroid co-cultures, as compared with control U373 in co-cultures by 50% and 40%, respectively (Figure 6B). The MMP-14 inhibitor reduced U373 cell invasion from co-cultured spheroids by approximately 35% below that of the U373 monospheroids. CA-074Me, MMP-9I and NSC405020 inhibitors reduced the invasion of MSCs from MSC/U373 co-cultured spheroids by 35%, 25% and 40%, respectively, but the inhibitors were not effective in MSC monospheroids (Figure 6B).

### Xenotransplantation of MSC/GBM cell mixtures into zebrafish embryo brain confirms differential behavior of U87 and U373 cells

**Figure 7:**
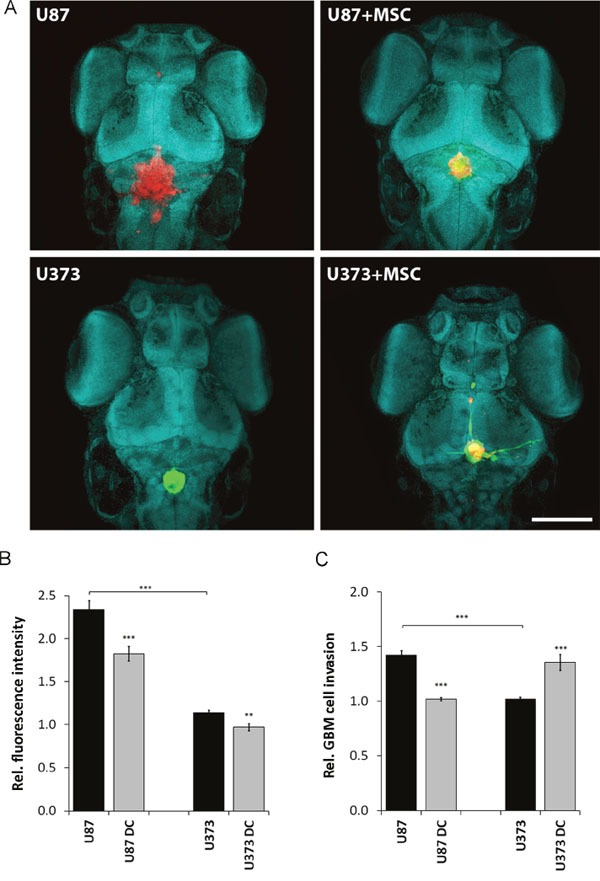
Proliferation and invasion of U87 dsRED and U373 eGFP cells in the zebrafish embryo brain upon co-injection with DiO/DiI- labelled MSCs **(A)** Two days after zebrafish embryo fertilisation, U87 and U373 cells alone (left upper and lower panels) or mixed with fluorescently stained MSCs with DiO (green) in the case of U87 (right upper panel), and with DiI (red) in the case of U373 (right lower panel), were injected into the brains of the zebrafish embryos. Cell nuclei were stained with methyl green (magnification, 10×, green blue shapes; scale bar = 250 μm). **(B)** GBM cell proliferation was determined 24 h and 72 after the injections by confocal microscopy and quantified as relative fluorescence intensity of U373eGFP and U87dsRed labelled cells injected alone or with MSCs (DC). **(C)** Relative invasion of U87dsRed and U373eGFP cells injected alone, or with fluorescently stained MSCs (DC) was determined as described in Material and Methods, clearly showing increased U373eGFP invasiveness and reduced U87 invasiveness from co-culture xenografts. Thirty zebrafish embryos were used per group. Data are means ± SD. ** P <0.01, *** P <0.001.

To confirm these results from *in vitro* assays *in vivo*, we injected GBM cells alone or together with MSCs into the brains of zebrafish embryos. Both U87 and U373 cells proliferated in zebrafish embryo brains (Figure 7A). However, after 72 h, fluorescence intensity of U87 cell xenografts was approximately 2-fold stronger compared to that of U373 cell xenografts, which implied more rapid proliferation of U87 cells in the zebrafish embryo brain (Figure 7B). Moreover, U87 cells invaded from the xenograft mass within the zebrafish embryo brain more readily than U373 cells. U87 cells showed 30% more relative invasion as compared to U373 cells (Figure 7C), which is in line with the *in vitro* observations. Co-injection of GBM cells and MSCs mixtures into the brain of zebrafish embryos resulted in reduced proliferation of both GBM cell types, in U87 cells by 20%, and in U373 cells by 15% (Figure 7B). Direct contact between MSCs and GBM cells enhanced the invasion of U373 cells by 35%, but inhibited invasion of U87 cells by 30% (Figure 7A, 7C), again similar to the findings *in vitro*.

### U87 and U373 cells differ in expression of genes involved in the cellular response to growth factor TGF-β

**Figure 8:**
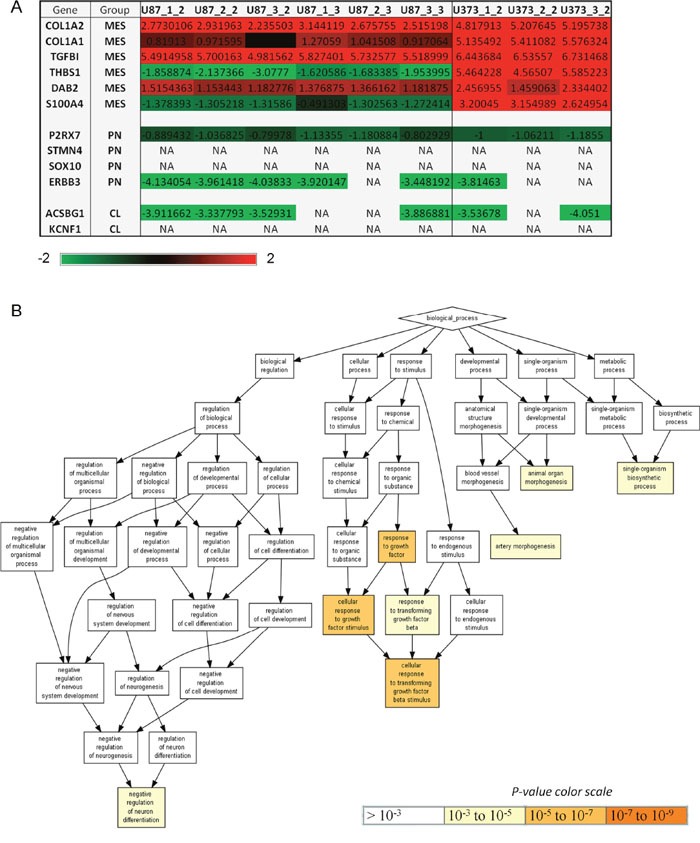
U87 and U373 cells differ in expression of mesenchymal genes and genes involved in response to growth factors, neuron differentiation, developmental and metabolic processes **(A)** Expression of 12 Behnan's genes [35] in U87 and U373 cells. These 12 genes (COL1A2, COL1A, TGFBI, THBS1, DAB2, S100A4, P2RX7, STMN4, SOX10, ERBB3, ACSBG1 and KCNF1) alone can subtype the GBM into 3 main subtypes: mesencyhmal (MES), proneural (PN) and classical (CL). Genes with the expression that was filtered out during quality control are denoted with NA. **(B)** Differentially expressed genes between U87 and U373 cells were functionally analyzed using Gene Ontology enRIchment anaLysis and visuaLizAtion tool (GOrilla) [36]. 294 genes with a significantly different standardized expression values between the two cell lines were inserted into GOrilla in order to determine the enriched pathways.

As U87 and U373 cells are both highly differentiated GBM cells lines, expressing low levels of GBM stem cells marker CD133, but phenotypically distinct [4] and showed differential response to MSCs, we were interested to investigate if these two cell lines show different GBM subtype according to *Verhaak and co-workers* [1]. Based on transcriptome analysis of 840 Verhaak's signature genes (210 genes per group) we observed that both, U87 and U373 cells, show mesenchymal signature (Supplementary Table 2). *Behnan et al*. [35] recently identified 12 genes, which could replace the 840 genes used for GBM genotyping and these genes also originate from Verhaak's classification. The analysis of these 12 genes revealed that U373 cells have stronger mesenchymal feature than U87 cells, as all the mesenchymal genes COL1A1, COL1A2, TGFBI, THBS1, DAB2 and S100A4 are highly expressed in U373 cells, but only some of these genes are also expressed in U87 cells (Figure 8A). Moreover, analysis using Gene Ontology enRIchment anaLysis and visuaLizAtion tool (GORILLA) [36] with differentially expressed Verhaak's genes in U87 compared to U373 cells showed that these two cell lines differ in the genes, which are associated with negative regulation of neuron differentiation, artery morphogenesis, animal organ morphogenesis and single-organism biosynthetic process. The most significant difference between these two cell lines was observed in the expression of genes involved in cellular response to growth factors and in particular in response to transforming growth factor beta (TGF-β) (Figure 8B). All the genes involved in response to TGF-β, such as COL1A1, ABL1, SOX9 and COL4A2 (Supplementary Table 3), were expressed more in U373 compared to U87 cells (Supplementary Table 2).

## DISCUSSION

Glioblastoma (GBM) cell heterogeneity is a consequence of the presence of different tumor and infiltrating stromal cells, among which the mesenchymal stem cells (MSCs) have not been studied to a great extent, compared to various types of immune cells. In addition to this non-cell-autonomous driver of (e.g., stromal) heterogeneity, the cell autonomous source of (e.g., genetic and epigenetic) heterogeneity [37], which relates to heterogeneous populations of GBM cells, may be present in a single tumor. The present study clearly demonstrates the intertwined relations of both kinds of heterogeneity *in vitro* and *in vivo*, showing opposite effects on invasiveness of two types of GBM cells by co-culturing them with bone marrow-derived MSCs.

Besides being part of the GBM microenvironment [28], MSCs have been suggested as therapeutic vectors for cellular therapy of GBM [23–30], but such treatment is still not approved due to observed opposite effects of MSCs on the same type of tumors *in vivo* and *in vitro* [28, 38]. However, here we show for the first time that the reason for the opposite effect is also the heterogeneity of GBM cells, highly relevant for an overall response of infiltrated and/or exogenously applied MSCs into the tumor. GBM U87 and U373 cells are frequently used in studies of various processes of GBM progression, but we only recently revealed that these cells show different GBM phenotype [4]. We also reported that bone marrow MSCs inhibited the invasion of indirectly co-cultured U87 cells [39], but increased invasion of U373 cells upon direct contact [24], and ascribed this obstacle to different co-culture conditions. Here, we used the same 3D spheroid assay of MSC/U87 and MSC/U373 co-cultures to study their invasiveness.

Invasion is defined as a three-step process: adhesion, induction of proteases, and migration via the loosened ECM [40]. This was later redefined by Friedl and Alexander [21], who dissected out the molecular processes that underlie various modalities of cell movement, showing that these are strongly depended on the tissue origin of the cells. MSCs strongly enhanced invasion of U373 cells, but not that of U87, on laminin, the main constituent of basal lamina in humans. Laminin is distributed around blood vessels and the pial surface (*glia limitans*), which have been reported as the preferential GBM migration pathways [10]. In contrast, MSCs strongly decreased U87 cell invasion in collagen I, which is the substrate most preferably invaded by these cells [12]. In spite of these matrix component preferences *in vitro*, differential invasion responses to MSCs was clearly cell-type-dependent. Diverse effects have been described for stromal fibroblasts that promoted invasiveness of breast cancer cells of the basal type, but not of the luminal type [41].

To confirm this differential effect of MSCs on GBM cells invasion in more physiological matrix, e.g. brain parenchyma *in vivo*, we used a novel animal model of zebrafish (*Danio rerio*) embryos. It is well known that most zebrafish organ systems are functionally similar to that of humans, and 82% of disease-causing human proteins have orthologues in zebrafish [31]. These zebrafish models have already contributed to a better understanding of a number of pathologies, including cardiovascular, neuronal and metabolic disorders [31], with respect to understanding their pathobiology and to study efficacy of therapy. As zebrafish embryos, do not develop adaptive immunity up to 3-4 weeks of age [32], they are replacing the NOD/SCID mice models of cancer progression, including for studying glioblastoma progression [34]. By using this animal system, we overcome the interfering effects of well recognized MSC immunomodulatory activity [22, 28, 42]. The zebrafish embryos’ brains mimic well the human GBM microenvironment with the presence of neuronal tracts and laminin at the early stages [43, 44], which justifies their use as a proper model for studying GBM invasion. Invasive GBM cells in the zebrafish embryonic central nervous system follow similar anatomical pathways as in human brains; *e.g*., along blood vessels [45] and axons [33, 34]. After their co-injection, we observed tight associations of MSCs and GBM cells in the growing mixed tumors in the brains of the zebrafish embryos, which suggested strong intercellular interactions. We demonstrated the same differential invasion responses of U87 and U373 cells to MSCs in zebrafish embryos as seen *in vitro*, confirming that the invasion pattern is indeed cell-type-specific, also within the more complex brain parenchyma matrix and microenvironment.

With respect of proteases, we show here that in MSC co-cultures of U373 cells, but not of U87 cells, the U373 invasion depended on the overexpression and activation of cathepsin B, which is a known as a potential initiator of the proteolytic cascade [14, 46]. Herein, the cathepsin B expression correlated with cell invasion, which was inhibited by its selective inhibitor CA-074. Cathepsin B is associated in the invasion process in many types of cancer [6] and these data confirmed the essential role of this cathepsin, in contrast to cathepsin L [47] and cathepsin K [7, 48] in GBM invasion. Interestingly, in the *in vivo* spheroid invasion assay of established GBM cell lines and primary GBM cultures from patients cathepsin B was highly expressed and only cathepsin B silencing and cathepsin B inhibitor, but not those of cathepsin L and S, inhibited GBM cell invasion in the collagen I embedded spheroids [49]. In NOD/SCID mice, cathepsin B was consistently located at the invasive edges of the GBM tumor [12]. Cathepsin B when translocated to newly formed invadopodia [12] anchors to the plasma membrane through the annexin II tetramer and may associates with uPAR [15] and α3β1 integrin to enhance invasion via FAK/Src kinase signaling [46]. Cathepsin B also activates uPA precursor, when this is bound to its membrane receptor uPAR [14].

Indeed, the MSC-enhanced invasion of U373 cells correlated well with increased levels of uPA/uPAR at the mRNA and protein levels. In contrast, the downregulated uPAR protein expression in co-cultured U87 cells correlated with their decreased invasion, in spite of increased expression of uPA. This confirmed that uPA activates an invasion cascade only upon binding to its receptor, as has been suggested previously [14, 50]. The uPA/uPAR complex is crucial in GBM invasion, as it is involved in ECM protein degradation, directly or indirectly via activation of plasminogen and pro-MMPs [50, 51]. Here we found increased amounts of MMP-2 and MMP-9 in the MSC/U373 co-culture medium that paralleled uPA/uPAR expression. Similar to U373 cells, activation of the uPA/uPAR-MMP axis was observed in direct co-cultures of fibroblasts with pancreatic carcinoma cells [52]. Moreover, calpains increase MMP expression and activity *via* glutamate-activated AMPA receptors and Ca^2+^ influx [16]. As we observed simultaneously increased secretion of calpain1 in MSC/U373 co-cultures, it is possible that calpain1 contributed to enhanced MMP-2/−9 levels and invasion. We functionally confirmed cathepsin B, MMP-9 and MMP-14 activation by the effects of their selective inhibitors, which completely abolished the effects of MSCs on enhanced U373 cell invasion, while having no effects on the co-cultured U87 cells. Taken together, we confirmed simultaneous activation of distinct proteases that may degrade ECM components and enhance U373 cell invasion.

In contrast, both GBM cell types enhanced invasiveness of MSCs out of the mixed spheroids, confirming our earlier results on U373 cell line [24]. However, this paralleled with a decrease of most protease levels in MSCs upon their direct contact with both types of GBM cells. It is possible that MSC invasion involves other proteolytic pathway(s), as the ones measured here, or proceeds by a protease-independent movement, possibly adopting a migratory phenotype, similar to an amoeboid movement [21].

Our last aim was to explore whether different response of U87 and U373 cells to MSCs is due to different GBM molecular subtype of these two established cell lines. When using the GBM molecular subtype classification according to *Verhaak et al*. [1] both, U87 and U373 cells belong to the mesenchymal subtype, although they significantly differ in expression of proteases and some other mesenchymal subtype-associated genes [35] as well as the genes, associated with cellular response to TGF-β. As observed, the identification of GBM subtype based on 4 Verhaak's gene expression fingerprints [1] is not sufficient to predict the response of GBM cells to stromal cells such as MSCs. Despite the fact that both, U87 and U373 cells have a predominant feature of mesenchymal GBM tumors, they react differently to MSCs. The reason may be due to fact that Verhaak's classification is based on heterogeneous GBM tissues, with respect to stromal and cancer cell components. Thus, this kind of classification is not enough adequate to characterize established cell lines that have been grown in serum conditions *in vitro* as single cell population.

Paracrine signaling between MSCs and GBM cells is mediated through growth factors and cytokines which are released from MSCs [22, 39]. Out of this, the most relevant with respect to invasion is TGF-β, which plays an important role in epithelial to mesenchymal transition [53] and shifts the expression of mesenchymal cell type-related genes in favor of more migratory/invasive GBM cell type [53, 54]. Additionally, TGF-β causes activation of invasion-related proteases, such as cathepsin B [55], MMP-2 and -9 [53]. We may speculate that exogenous TGF-β, released from MSCs, was triggering the proteases expression, increasing U373 invasion, but that was not the case in U87 cells. This may be due to TGF-β triggering alternative signaling pathways [56], in the two phenotypically different GBM cell lines. The reason for differential U87 and U373 cells’ response in the same (MSC mediated) microenvironment may also/in addition dwell in their diverse cytokines’ expression [4], as for instance, compared to U87 cells, the U373 express and secrete more CCL2/MCP-1 [4], which is a known activator of gelatinases MMP-2 and MMP-9 *via* ERK 1/2 signaling [57].

Taken together, we have demonstrated specific responses of these two GBM cell phenotypes to MSCs both *in vitro* and *in vivo*. We have shown that MSCs reduce the invasion of U87 cells, but enhance the invasion of U373 cells, which is mediated by upregulated cathepsin B, calpain1, uPA/uPAR, as well as MMP-2, -9 and -14 in this cell line. Moreover, we showed that differentially expressed genes, associated with cellular response to TGF-β, in U87 and U373 cells may be responsible for differential behavior of these two cell lines in co-cultures. By monitoring GBM cell invasion upon their co-injection with MSCs into the brains of zebrafish embryo, we confirmed these GBM cell-type-dependent responses to MSCs also *in vivo*. We demonstrated that zebrafish embryos are an excellent model for addressing GBM intra-tumoral heterogeneity, which opens new avenues for studying GBM microenvironment. However, the relevance of these data *in vivo*, e.g. in the presence of various GBM infiltrating cells of immune system, such as T-cells and natural killer cells, is questionable. Multilateral interactions, such as for example between GBM/T-cells/NK cells [58] and MSC/NK cells, may namely put the presented data in rather different perspective.

## MATERIALS AND METHODS

### Cell culture

Human bone marrow-derived MSCs were obtained from Lonza Bioscience (Lot. 6F4393, USA) and were cultured according to the manufacturer recommendations [30]. Human GBM cell lines (U87, U373 cells) were obtained from American Type Culture Collection (USA), and all cell types were cultured in the same growth medium. Authentication of the cell lines was performed as described previously [7]. U373 cells stably expressing enhanced green fluorescent protein (U373 eGFP cells) [24] and U87 cells expressing red fluorescent protein (dsRED) were prepared as described previously [59].

### Preparation of monolayer and spheroid co-cultures

Monolayer co-cultures of MSCs and GBM cells (U87 or U373) were prepared by mixing the cells in three different ratios (MSC/GBM cells) of 1:3, 1:1 and 3:1, that were seeded into monolayer culture plates with formats, corresponding to each particular experiment. The cells were analyzed after 72 h of direct co-culturing.

For monitoring of invasion of MSCs and GBM cells in MSC/GBM spheroid co-cultures, MSCs were labeled prior to spheroid formation with the fluorescent dyes Vybrant DiO/ DiI (Molecular Probes, USA), according to the manufacturer instructions. For mixed spheroid formation, Vybrant-labelled MSCs and U87 dsRED or U373 eGFP cells were mixed in a 1:1 ratio and seeded in medium containing 4% methylcellulose in U-bottomed 96-well plates (2.5 ×10^3^ cells/well; BD Biosciences, USA), which were then centrifuged at 850×*g* for 90 min, and then incubated overnight.

### Three-dimensional spheroid invasion assays

For the assessment of cellular invasion, fluorescent MSCs and GBM cells and mixed spheroids were transferred into 24-well plates (Corning, Life Sciences, USA) and embedded in collagen type I (1 mg/mL; BD Biosciences) or Matrigel (6.35 mg/mL; BD Biosciences). To assess cellular invasion on laminin, the 96-well plates were coated with laminin (2 μg/cm^2^; Sigma-Aldrich). The spheroids were then placed in the middle of each well in the growth medium. Cell invasion was monitored using a fluorescence inverted microscope with the NIS-Elements software (Eclipse Ti-E; Nikon, Japan). Invasiveness was defined as the distance measured from the edge of the spheroids to the most distant cell population, divided by the spheroid diameter.

### Transcriptome analysis

To select candidate proteases for *in vitro* analysis the transcriptome analysis of GBM tissue and normal brain tissue was performed as described previously [7]. Briefly, raw data of gene expression for GBM tissue and normal brain were taken from The Cancer Genome Atlas data portal (July 2011; https://cancergenome.nih.gov/) as Batch 8 (24 GBM samples, 10 unmatched control brain samples). As these data were merged from different experimental sources, a Bioconductor package of RankProd was used for the detection of differentially expressed genes. Differentially expressed genes with their percentage of false predictions <0.05 that have been recently identified for GBM tissue *versus* normal brain tissue were published recently [7]. Among the upregulated genes, we used unique Entrez IDs to search for the intersections with a list of human proteases (653) obtained from the MEROPS database that were selected in October 2013.

The transcriptome analysis of U87 and U373 cell lines was performed as described previously [39]. Briefly, six biological replicates of U87 cells and three biological replicates of U373 cells were used. Samples were hybridized to Illumina HumanWG-6 v3 Expression BeadChip (Illumina BeadChip; Illumina, San Diego, CA, USA). After scanning, image acquisition was carried out by applying BeadStudio version 3.3.7 software (Illumina). Data is deposited in NCBI's Gene Expression Omnibus (GEO, http://www.ncbi.nlm.nih.gov/geo/) – series GSE26283 (samples GSM645515, GSM645519 and GSM645523) for U87-MG cells and series GSE59634 (samples GSM1440969, GSM1440973 and GSM1440977) for U373 cells. Data preprocessing was done as described in *Verhaak et al*. [1]. Data were log-transformed and median centered. The 840 genes that were defined as marker genes for classification of GBM samples into one of the four groups (MES – mesenchymal, PN – proneural, N – neural and CL – classical) by Verhaak were then filtered out and sorted (see Supplementary Table 2). Only genes that passed the quality control were taken into consideration (517 genes overall). The genes with the high standardized gene expressions were color-coded in red, while genes with low standardized gene expressions were colored in green. The highest number of highly expressed genes for both U373 and U87 cell lines corresponded to the MES subtype (as determined by *Verhaak et al*. and shown in the column B on Supplementary Table 2). Finally, we used t-test in order to determine the genes with a significantly different expression values between the two cell lines (U373 and U87). The resulting 294 genes were then uploaded to GOrilla (http://cbl-gorilla.cs.technion.ac.il/) [36] to identify and visualize the enriched pathways, i.e. the pathways that differ between the both cell lines in terms of gene ontology.

### qRT-PCR

Total RNA was isolated from MSCs, GBM cells and their direct co-cultures (as monolayers) using Trizol reagent (Invitrogen, UK). cDNA was generated from 1 μg total RNA using High Capacity cDNA Reverse Transcription kits (Applied Biosystems, USA). The expression of candidate protease genes (Supplementary Table 4) was quantified using real-time quantitative PCR (ABI 7900 HT Sequence Detection System). Real-time PCR reactions were performed using 1:5 dilutions of each cDNA, added to TaqMan Universal PCR Master Mix and TaqMan Gene Expression assays (Applied Biosystems). The analyses were performed with the SDS v2.2 software (Applied Biosystems, USA), and the mRNA levels relative to the housekeeping gene GAPDH mRNA are presented.

### Flow cytometry

After 72h of co-culturing the mono and direct co-cultures of MSCs and GBM cells (U87 dsRED and U373 eGFP) were collected and pelleted. Cell pellets were washed by 1x PBS, resuspended and fixed in 0.5% gluteraldehyde. Permeabilization was performed with 0.1% Triton X-100. Cells were incubated for 30 min with the primary antibodies. Primary antibodies and their dilutions are listed in Supplementary Table 5. After washing with 1x PBS the secondary anti-mouse or anti-rabbit antibodies were used (1:300; conjugated with Alexa Fluor 488 for U87 dsRED co-cultures, or Alexa Fluor 647 for U373 eGFP co-cultures; Life Technologies, UK) and incubated for 30 min. After final PBS wash, the cells were re-suspended in 300 μl of 1x PBS and analyzed by flow cytometer (BD FACSCalibur, BD Biosciences) using the CellQuest software (BD Biosciences). To distinguish between proteases expressed in MSCs and fluorescent GBM cells and to show expression of protease in different cell populations, cell gating based on fluorescence was performed. Protein expression of candidate protein in each cell type was determined as percentage of cells stained positive for candidate protein out of total (stained and unstained) cells (see Supplementary Method).

### Western blotting

Western blots were performed with conditioned medium from monocultures and direct MSC/GBM co-cultures grown as monolayers. After 72 h of co-culturing, the culture media were replaced by serum-free medium. The conditioned media was centrifuged at 300×*g* for 10 min, and concentrated 100-fold using an Amicon Ultra-4 centrifugal filter device with a 10-kDa cut off (Merck Millipore, Germany), according to the manufacturer's instructions. Concentrated media (15 μg total protein) were subjected to Western blotting [7] using the indicated antibodies (Supplementary Table 5).

### Cytotoxicity assay

To determine cellular toxicity of synthetic protease inhibitors and the final non-toxic concentrations for use in further experiments, MTT assays were performed. MSCs, U87 and U373 cells were plated into 96-well plates and left to grow for 24 h before addition of the following cell-permeable protease inhibitors (dissolved in dimethylsulphoxide [DMSO]), and with 0.1% DMSO as control: CA-074Me, a cathepsin B inhibitor (Peptide Institute, Japan), MMP-9 inhibitor I (Calbiochem, USA), and NSC405020, a MMP-14 inhibitor (Tocris Bioscience, UK). After 72 h incubation, the MTT reagent (Sigma-Aldrich) was added to the cells, and the formazan crystals formed were dissolved in DMSO. Absorbance was measured at 570/690 nm with a plate reader (Synergy MX; BioTek, USA).

### Effects of protease inhibitors

Spheroid invasion assays were set up with fluorescent MSCs, GBM cells, and mixed co-culture spheroids on laminin-coated 96-well plates, as described above. The selective synthetic protease inhibitors were used at their non-cytotoxic final concentrations: 2 μM CA-074Me, 100 nM MMP-9 inhibitor I, and 10 μM NSC405020, all diluted in 0.1% DMSO. Cell invasion was assessed after 72 h, as described above.

### Zebrafish embryo model

Wild-type AB zebrafish (*Danio rerio*) embryos were collected and incubated at 26°C in dilution water (ISO 7346-3:1996). After 36 h of age 0.005% phenylthiourea was added to the water to inhibit pigment formation. Zebrafish embryo xenotransplantation experiments were performed as described previously [34]. Prior to implantation, MSCs were labeled with Vybrant DiI or DiO (Molecular Probes, USA) for co-implantation with the U373 and U87 cells, respectively. Mixtures of GBM cells with labeled MSCs were prepared in a 1:1 ratio. Xenotransplantation of 50 to 100 GBM cells or 100 to 200 cells of the MSC/GBM mixture (containing 50-100 GBM cells) was performed by injecting 5 nl of cell suspension into the brain of embryos at 52 h after fertilization with the MICROINJECTOR system (Tritech Research, USA). Embryos with implanted cells were incubated at 31°C. For quantification of GBM cell invasion, fluorescence images of GBM cells in the embryos in lateral orientation were obtained at 1 day and 3 days after cell implantation, using an inverted fluorescence microscope (Eclipse T300; Nikon, Japan). The largest diameter and the diameter perpendicular to the largest of each xenograft were measured in images, and these two diameters were then averaged to obtain a parameter of xenograft size as a measure of cell invasion. Relative GBM cell invasion was compared between GBM cells alone and the MSC/GBM mixed xenografts, using the Welch's t-test. To determine differences in proliferation rates of GBM cells in MSC/GBM mixed xenografts, relative changes in fluorescence intensity of GBM cells between 1 day and 3 days after cell implantation were quantified using ImageJ (http://rsbweb.nih.gov/ij/; National Institutes of Health, USA). For confocal imaging, the embryos were fixed at 72 h after cell injection using 4% paraformaldehyde in phosphate-buffered saline for 2 h, and cleared in Sca*l*e U2, which was modified to enable visualization of carbocyanine-dye-labelled cells [60]. Imaging was performed with a TCS SPE confocal microscope (Leica) as described previously [34].

### Statistical analysis

Unless stated otherwise, statistical analysis was performed with one-way ANOVA, followed by the Bonferroni tests for pair-wise comparisons, in GraphPad Prism. P <0.05 was considered to indicate statistically significant differences. Data are expressed as means ± standard deviation (SD) of 3 independent experiments, with each performed at least in duplicate.

This work was supported by the Operational Program for Italy-Slovenia, 2007–2013 (the INTERREG project) CB134-GLIOMA, the ARRS project J1-4274, and ARRS Program P10245 (all to TTL), and the ARRS Young Researcher grant to BB.

## SUPPLEMENTARY MATERIALS FIGURES AND TABLES


