# Supplementary material for: Mesenchymal stem cells differentially affect the invasion of distinct glioblastoma cell lines
Source: Oncotarget. 2017 Mar 9;8(15):25482–99. doi: 10.18632/oncotarget.16041 (PMC5421945; doi:10.18632/oncotarget.16041)
Supplement: Supplementary file 1 [file oncotarget-08-25482-s001.pdf]

Mesenchymal stem cells differentially affect the invasion of distinct glioblastoma cell lines

SUPPLEMENTARY MATERIALS

Flow cytometry analysis

Protein expression of candidate protein in each cell type was determined as percentage of cells stained positive for candidate protein out of total (stained and unstained) cells. Example of flow cytometry analysis and calculation of MMP-14 expression in co-cultured U373 eGFP cells and MSCs after 3 days in co-cultures:

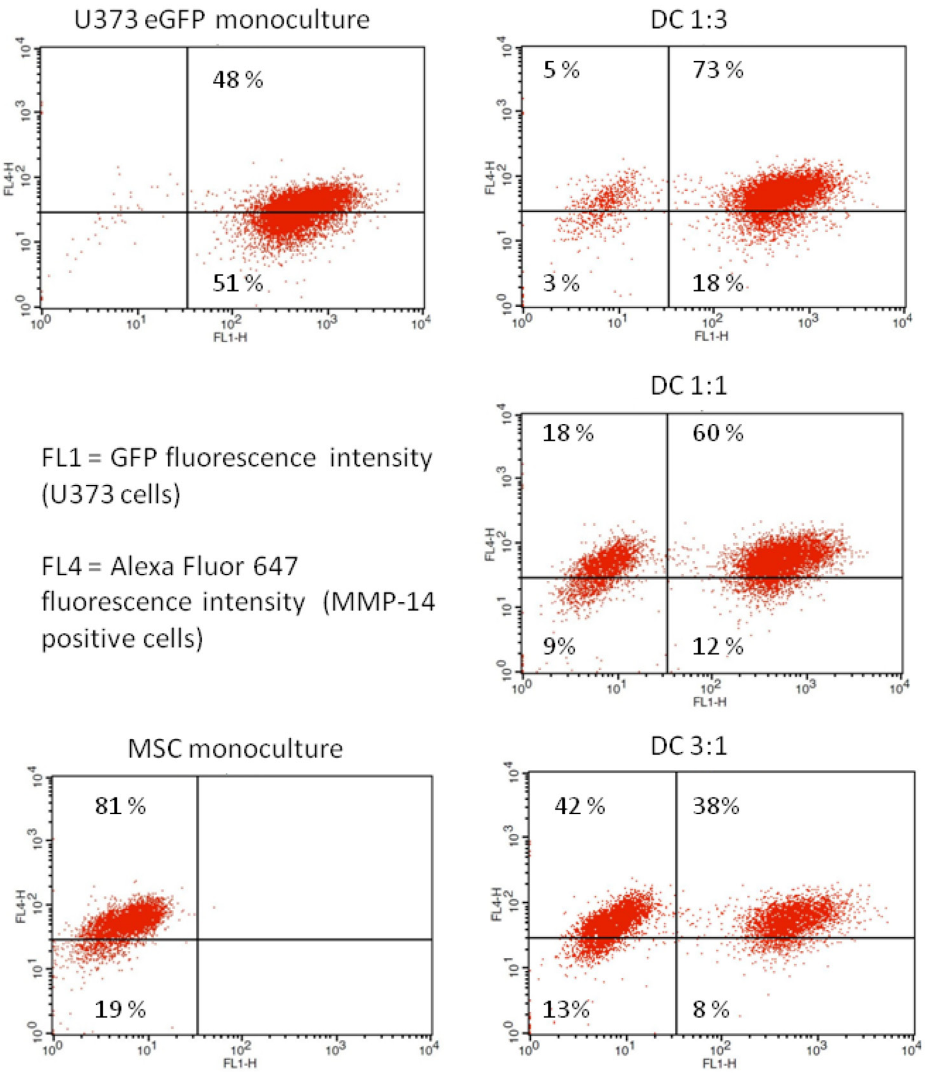

MMP-14 expression in U373 cells and MSCs in DC 3-1 co-culture:

% protein expression in U373 cells = **38/(38+8)= 82,6 %**

% protein expression in MSCs = **42/(42+13)= 76,4 %**

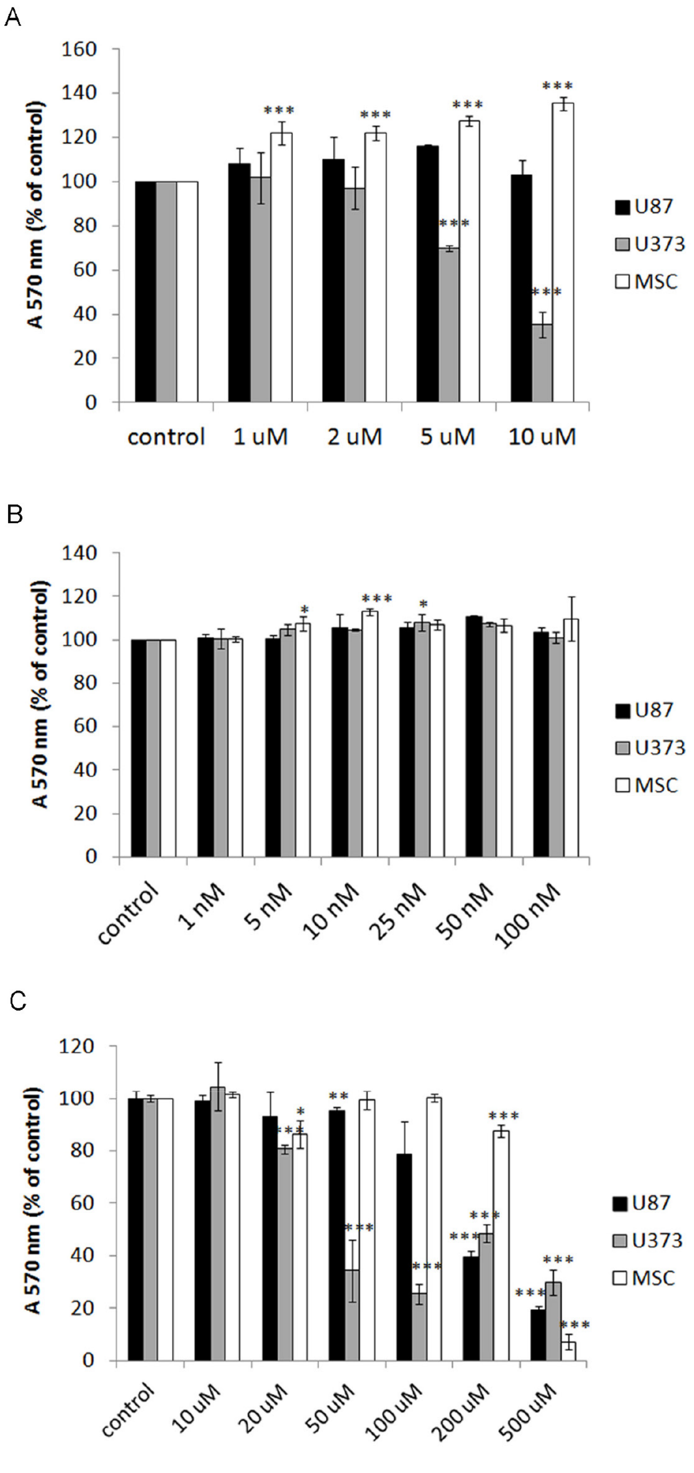

Supplementary Figure 1: MSC and GBM (U87 and U373) cell viability in the presence of the selective protease inhibitors CA-074Me (A), MMP-9 I (B), and NSC405020 (C). Cells were plated and incubated with series of concentrations of protease inhibitors during 72 h. Cell viability was determined using MTT assays as absorbance **(A)** at 570 nm relative to the control (0 nM/μM of inhibitor). The inhibitor concentrations were considered non-toxic when cell viability was >95% as compared to the control. Data are means ± SD. * P < 0.05, ** P < 0.01, *** P < 0.001.

Supplementary Table 1: Upregulated protease genes in GBM tissue, as compared to normal brain

See Supplementary File 1

Supplementary Table 2: Expression of genes from TCGA 840 gene list [1] in U87 and U373 cells

We analyzed 6 biological replicates of U87 and 3 biological replicates of U373 cells as described in Material and Methods section. Based on the data pre-processing as done in *Verhaak et al*. [1], 517 genes were used for determination of GBM subtype: PN – proneural, NL – neural, CL – classical and MES – mesenchymal (as determined by *Verhaak et al*., column B). Upregulated genes are shown in red. The mesenchymal subtype (MES) had the most upregulated genes in both, U87 and U373 cell lines. Genes with the expression that was filtered out during quality control are denoted with NA.

See Supplementary File 2

Supplementary Table 3: GO enrichment results for the 294 genes with significantly different expression levels between U87 and U373 cell lines

The enrichment p-value and the FDR corrected p-value, together with the enrichment values used for the p-value calculation using the hypergeometric model are shown.

See Supplementary File 1

Supplementary Table 4: List of TaqMan probes (Applied Biosystems) used in the qRT-PCR analyses

GENE SYMBOL	GENE NAME	TAQMAN PROBE
CAPN1	Calpain1	Hs00559804_m1
CAPN2	Calpain 2	Hs00965097_m1
CTSB	Cathepsin B	Hs00947433_m1
MMP2	MMP-2	Hs001548727_m1
MMP9	MMP-9	Hs00234579_m1
MMP14	MMP-14	Hs00237119_m1
PLAU	Urokinase-type plasminogen activator (uPA)	Hs01547054_m1
PLAUR	Urokinase-type plasminogen activator receptor (uPAR)	Hs00958880_m1
GAPDH	Glyceraldehyde 3-phosphate dehydrogenase	Assay No. 4310884E

Supplementary Table 5: List of primary antibodies and their dilutions used in the flow cytometry analysis and western blotting

PRIMARY ANTIBODY	SOURCE	Flow cytometry	Western blotting
**Rabbit anti-calpain 1**	Abcam (ab28258)	1:200	1:500
**Rabbit anti-calpain 2**	Abcam (ab39165)	1:100	1:100
**Rabbit anti-cathepsin b**	Krka, d.d.	1:10	1:200
**Mouse anti-mmp-2**	Abcam (ab3158)	1:20	1:200
**Rabbit anti-mmp-9**	Abcam (ab38898)	1:100	1:1000
**Rabbit anti-mmp-14**	Abcam (ab3644)	1:20	/
**Rabbit anti-upa**	Abcam (ab24121)	1:100	1:200
**Rabbit anti-upar**	Abcam (ab103791)	1:100	/
**Mouse isotype control IgG1 κ**	Abcam (ab91535)	1:10	/
